# Optimize the Coverage Probability of Prediction Interval for Anomaly Detection of Sensor-Based Monitoring Series

**DOI:** 10.3390/s18040967

**Published:** 2018-03-24

**Authors:** Jingyue Pang, Datong Liu, Yu Peng, Xiyuan Peng

**Affiliations:** School of Electrical Engineering and Automation, Harbin Institute of Technology, Harbin 150080, China; jypang@hit.edu.cn (J.P.); pengyu@hit.edu.cn (Y.P.); pxy@hit.edu.cn (X.P.)

**Keywords:** satellite, anomaly detection, coverage probability, prediction interval, Gaussian process regression, relevance vector machine

## Abstract

Effective anomaly detection of sensing data is essential for identifying potential system failures. Because they require no prior knowledge or accumulated labels, and provide uncertainty presentation, the probability prediction methods (e.g., Gaussian process regression (GPR) and relevance vector machine (RVM)) are especially adaptable to perform anomaly detection for sensing series. Generally, one key parameter of prediction models is coverage probability (CP), which controls the judging threshold of the testing sample and is generally set to a default value (e.g., 90% or 95%). There are few criteria to determine the optimal CP for anomaly detection. Therefore, this paper designs a graphic indicator of the receiver operating characteristic curve of prediction interval (ROC-PI) based on the definition of the ROC curve which can depict the trade-off between the PI width and PI coverage probability across a series of cut-off points. Furthermore, the Youden index is modified to assess the performance of different CPs, by the minimization of which the optimal CP is derived by the simulated annealing (SA) algorithm. Experiments conducted on two simulation datasets demonstrate the validity of the proposed method. Especially, an actual case study on sensing series from an on-orbit satellite illustrates its significant performance in practical application.

## 1. Introduction

With the development of sensing and acquisition technology, more sensing data series of system condition are available. Data mining and knowledge discovery of these sensing data series can help mine the contained fault or failure information [1,2]. For practical application, one of the most valuable strategies is to detect the data which behave differently from the majority. The detected data are defined as anomalous in the domain of machine learning [3]. Anomaly detection is also the problem of finding items, events, or observations that do not conform with an expected pattern or a model of normal behavior [4]. The application fields of anomaly detection include network intrusion detection [5], financial fraud detection [6], medical sensor detection [7], and fault detection in industrial systems [8], etc. For system condition monitoring, the detected anomalous data can be excluded to prevent incorrect decision making. Especially, in the area of aeronautics and astronautics, the system reliability and operation safety can be enhanced by anomaly detection among telemetry series (i.e., sensing data series).

There are now three broad categories of anomaly detection techniques based on the availability of labels [4]. When a training dataset contains both normal and outlying instances, a supervised learning approach referring to a standard classification algorithm can be established to detect anomalies [9]. However, in most applications, the accumulated anomalous samples are generally insufficient and inaccurate, which brings very challenging issues to the supervised methods. To address this, some semi-supervised anomaly detection techniques are applied to model the normal records, and only the records that do not comply with the generated model are labeled as anomalous. Semi-supervised methods of anomaly detection mainly refer to one-class classification methods (e.g., one-class support vector machine (OC-SVM) and support vector data description (SVDD) [10,11]) and prediction-based methods (e.g., least squared support vector machine (LS-SVM) [12], relevance vector machine (RVM) [13], Gaussian process regression (GPR) [14], and artificial neural network [15]). The third type of learning is unsupervised. In detail, the input of an unsupervised method has no labeling information, and only a small fraction of the data samples is outlying [16]. Nearest-neighbor-based methods are the most widely applied for unsupervised leaning [17]. It must be taken into consideration that normal samples are generally accumulated easily but anomalous samples are much rarer in industrial areas. Furthermore, simulation is expensive and incomplete. Thus, the semi-supervised methods are our focus.

The performance of one-class classification methods significantly depends on the set length of the time window. While these prediction-based methods label the samples based on the differences between the predicted values and the observed ones [18], it is more flexible to detect the anomalies with unknown length. Among these prediction methods, some point prediction methods (e.g., Naïve Bayes, LS-SVM, multi-layer perceptron (MLP), nearest cluster (NC) predictor, and single-layer linear network (LN) predictor) have been combined with K-fold cross-validation or observation error estimation to realize anomaly detection [19,20]. Compared with these point predictions which must perform extra operation or combine other methods to construct the prediction interval (PI), the prediction models with uncertainty presentation (e.g., GPR and RVM) are more suitable for detecting anomalies. These models can provide both the point prediction and interval estimation [21,22], then the points beyond the corresponding PIs will be labeled as abnormal. Therefore, this paper focuses on GPR and RVM models.

GPR and RVM models describe a new sample by mean and variance of normal distribution under the Bayesian framework. By comparison, RVM is a sparse model with higher efficiency in the testing phase, while the training is time-consuming [19]. When the prediction model is constructed with the available training data set, the normal range of a new sample is estimated by PI with the setting coverage probability (CP), where CP is the probability that the target lies within the provided PI. From a statistical point of view, the CP is generally set to 90% or 95%, which can cover normal data in a relatively acceptable probability of making type I error and type II error. Especially for prediction and anomaly detection, 90% and 95% are also the regular set values [23,24,25]. For normal distribution, the CP of 95% corresponds to the PI of [u−2σ,u+2σ], where *u* and σ are the mean and standard variance, respectively. Obviously, the PI with a higher CP setting generally meets the potential challenges of missing rates. Conversely, the smaller CP setting has a narrower PI, which may cause some false alarms. In other words, the width of PI and the detection performance are contradictory. Therefore, it is very meaningful to estimate the performance of PI with different CPs and realize better anomaly detection with the optimal PI. It is noted that the concept of CP also appears in heterogeneous wireless cellular networks (HWCNs), and it refers to how many mobile users can reliably receive data from a base station in a practical HWCN over the test users, which is different from our focus [26,27,28,29]. To realize better prediction, some methods are designed to choose a CP [30,31] for multi-step prediction. However, they are different from our research background that realizes anomaly detection based on one-step prediction. According to the present research, there are few criteria to select the optimal CP for prediction-based anomaly detection. 

The receiver operating characteristic (ROC) curve offers a graphical illustration of these trade-offs between sensitivity (true positive rate) and specificity (true negative rate), and has been used for the determination of an “ideal” cut-off value [32]. However, it must be noted that the anomalous samples are always insufficient to compute the specificity in the training step, which makes the ROC curve less effective in estimating the performance of the PIs with different CPs. Therefore, this paper designs a graphic indicator of receiver operating characteristic of PI (ROC-PI) on the basis of the ROC curve.

In detail, ROC-PI offers a graphical illustration of these trade-offs between PI coverage probability (PICP) and the width of PI. PICP is also called the PI confidence level, and is the probability that the testing targets lie within the PI provided by one prediction model. The width of the PI is represented by the CP of the prediction distribution. Moreover, three criteria (i.e., points on the ROC curve closest to (0, 1), the Youden index, and the minimized cost criterion) have been developed to optimize the threshold point of the ROC curve [33]. For the ROC curve, (0, 1) is the ideal case for anomaly detection, so the point on the ROC curve closest to (0, 1) is optimal. Nevertheless, for the ROC-PI curve, the point (0, 1) means the CP of PI is not consistent with the PICP, which is unrealistic in real application. Thus, the effective point like (0, 1) is difficult to determine which makes the criteria (i.e., points on the ROC curve closest to (0, 1)) inappropriate for seeking the optimal point in the ROC-PI. The Youden index maximizes the vertical distance from the diagonal line. Namely, the Youden index is the point on the ROC curve with the farthest distance from line of equality (diagonal line). Moreover, the Youden index is more generally used with the advantage of reflecting the intension to maximize the correct classification rate and is easy to calculate [34]. The third criterion considers cost and is rarely applied because it is difficult to implement. Given the properties of ROC-PI, the point of diagonal reflects the effective estimation of prediction distribution whose CP is equal to the PICP, so the point on the ROC-PI curve closest to the line of equality is optimal within an acceptable range of CP. Namely, the optimal CP can be calculated by the modified Youden index. In addition, considering that the simulated annealing (SA) method has been utilized to solve this type of optimization problem [35,36,37], the Youden index is modified in this paper to determine the optimal CP based on the SA method. 

On this basis, an improved method for anomaly detection with a probability prediction model is realized in this work. It is noted that the proposed method is not only suitable to GPR and RVM models, but can also apply to other probability prediction models, which can provide the distribution of new testing data. GPR and RVM are two typical probability prediction models, and they have different advantages: RVM is a sparse model which can give a quick testing result, while GPR is a non-parametric model which can be trained quickly and flexibly. Therefore, in order to test our proposed method comprehensively, both of them are considered to be the testing models in this work. The experiments on the simulated data and real spacecraft telemetry series validate the effectiveness and applicability of the proposed method.

## 2. Sensing Data Anomaly Detection Framework with Prediction Model

### 2.1. Anomaly Detection Based on Prediction Interval

**Figure 1:**
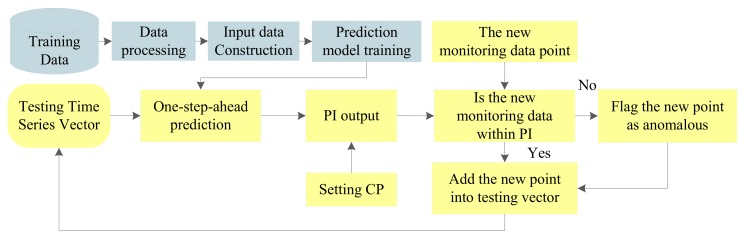
Anomaly detection based on prediction interval (PI).

In statistical inference (specifically predictive inference), a PI is an estimate of an interval within which one or more future observations will fall with a certain probability given what has already been observed. A confidence interval only provides bounds for a scalar population parameter, such as the population mean [38]. By way of comparison, a PI contains the noise interference with the injected noise variance. Therefore, the PI is more effective for anomaly detection. Figure 1 shows its framework.

In Figure 1, the important steps are data preprocessing, input data construction, prediction model training, and PI output.

1. Data preprocessing. In this step, the erroneous data points are deleted and data normalization is performed by statistical analysis and min-max normalization, respectively. Then, the signal amplitude is restricted to the range from −1 to 1. It is noteworthy that some preprocessing methods related to some specific areas can also be performed in this step.
2. Input data construction. In this work, autocorrelation analysis is applied to obtain the embedding dimension to construct the input matrix.
3. Prediction model training. In this step, the initial model parameters and the optimization algorithms are determined first, then the prediction model is constructed based on training data.
4. PI output. Combined with the sample distribution estimated by the one-step-ahead prediction model and the CP setting, a PI is constructed to reflect the normal range of a new monitoring point. The CP is set by default to, e.g., 90% or 95%.

**Figure 2:**
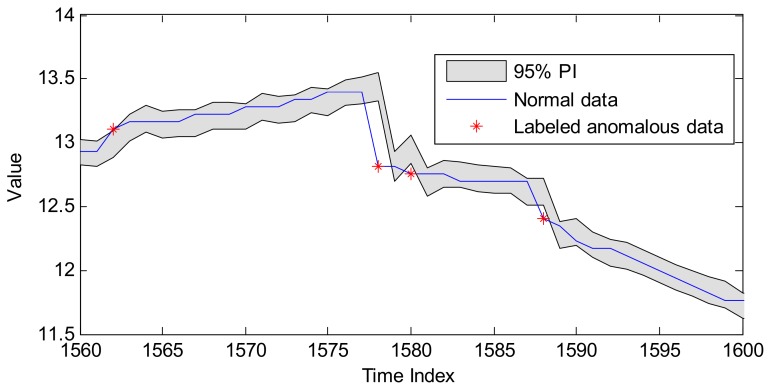
An example of anomaly detection with PI.

Based on the above steps, a PI is constructed as the threshold to judge whether the detected point is normal or abnormal. Given that the predicted models with uncertainty presentation can provide the PI directly with the data distribution estimation, they are very suitable for the anomaly detection of time series (in this work, time series indicate the sensing data series). An example of anomaly detection with PI is shown in Figure 2.

In Figure 2, the grey region is the 95% PI provided by the GPR model, and the four points beyond the corresponding range will be labeled as anomalies. In this paper, two prediction models with uncertainty presentation (i.e., GPR and RVM) are applied to perform anomaly detection, which will be introduced in the following two subsections.

### 2.2. Prediction Interval Estimation Based on Gaussian Process Regression

GP defines a collective of random variables where the combination of any finite dimensional variables obeys a joint Gaussian distribution [22]. Compared with Gaussian distribution for a single random variable whose properties are represented by mean and variance, mean function and covariance function are the characteristics of Gaussian process defined by Equations (1) and (2), respectively.

(1) m(x)=E[f(x)],

(2) $$k(x_{i},x_{j})=E[(f(x_{i})-m(x_{j})(f(x_{i})-m(x_{j}))],$$

where *E*[ ] is the Expectation function. $x_{i}$ and $x_{j}$ are different input variables. $k(x_{i},x_{j})$ reflects the relation between $x_{i}$ and $x_{j}$. The most-used covariance function is the square exponential function [39]:

(3) $$k(x_{i},x_{j})=\upsilon_{0}\exp\left\{-\frac{1}{2}\sum_{l=1}^{d}\omega_{l}{\left(x_{i}-x_{j}\right)}^{2}\right\},$$

where $\upsilon_{0}$ and $\omega_{1},\omega_{2},\ldots ,\omega_{d}$ are hyper-parameters which need to be initialized. $\upsilon_{0}$ is model variance, and $\omega_{l}$ is the distance size. It is noted that users can define the covariance function as long as it meets the nonnegative conditions. Generally, based on the normalization of input variables, the mean function can be set to zero everywhere. In this case, the prior distribution of GP is determined by the covariance function as well as its set hyper-parameters. In practical applications, these initial hyper-parameters can be set randomly, ranging from 0 to 1. Moreover, conjugate gradient method is adopted to optimize these hyper-parameters.

Given the regression problem defined by the following equation:

(4) y=f(x)+ε,

where ***x*** is *d* dimensional input variables and ***y*** is the target variable, f(x) describes the functional relationship between ***x*** and ***y***. ε is supposed to be additive white noise.

Some parametric models restrict the explicit form of f(x) with some unknown parameters. However, the GPR model just assumes that the function values $f(x_{1}),\ldots ,f(x_{N})$ with different input variables obey a joint Gaussian distribution, then $f(x_{1}),\ldots ,f(x_{N})$ forms a GP described as Equation (5):

(5) $$f(x)\sim GP(m(x),k(x_{i},x_{j})).$$

One important property of GP is described by Definition 1.

**Definition** **1.** *The sum of two independent multivariate normal distributions (e.g., A and B) is also a multivariate normal distribution (e.g., C), whose mean and variance are both the sum of the mean and variance of A and B*.

Based on the property of GP described by Definition 1, also with Equations (4) and (5), the target ***y*** obeys a GP:

(6) $$y\sim GP(m(x),k(x_{i},x_{j})\;+\sigma_{n}^{2}\delta_{ij}\;),$$

where $\delta_{ij}$ represents the Dirac function, $\delta_{ij}=1$ only when i=j.

Then, suppose $f_{*}$ is the function variable at one test input $x_{*}$. Multiple-tests are also allowed. ***y*** and $f_{*}$ still obey a joint Gaussian distribution (still based on the above property of GP), namely:

(7) $$\left(\begin{array}{l} y\\ f_{*} \end{array}\right)\sim \left(\left[\begin{array}{c} m\\ m_{*} \end{array}\right],\left(\begin{array}{cc} A & E\\ E^{T} & B \end{array}\right)\right)\;,$$

where ***m*** is the mean vector of training data, and $m_{*}$ is the mean vector of testing data. In addition, ***A*** is the covariance matrix constructed by the training set itself, which also considers the noise variance, $A(i,j)=k(x_{i},x_{j})+\delta_{n}{}^{2}$, $A\in R^{N\times N}$. *N* is the training size. ***E*** is the covariance vector of the training set with testing input, $E(i)=k(x_{i},x_{*})$. Similarly, *B* is the covariance value of the testing input itself, $B=k(x_{*},x_{*})$. 

Another important property of GP is described by Definition 2.

**Definition** **2.** For a multivariate normal distribution (e.g., C) constructed by two multivariate normal distributions (e.g., A and B), when a part of the observed value (e.g., C_1_) is known, the probability distribution of another part of the observed value (e.g., C_2_) is also a multivariate normal distribution whose property can be expressed by the corresponding information of A and B.

Based on Definition 2 and Equation (7), the marginal distribution of ***y*** can be derived as Equation (8), and the condition distribution of ***y*** with known $f_{*}$ is given by Equation (9).

(8) y~N(m,A),

(9) $$y\;|\;f_{*}\sim N(m+EA^{-1}(f_{*}-m_{*}),B-EA^{-1}E^{T}),$$

where *N*(.,.) represents a joint Gaussian distribution. Therefore, Equation (8) indicates that ***y*** obeys the joint Gaussian distribution with mean vector ***m*** and covariance matrix ***A***, so as Equation (9).

Then the posterior conditional distribution of $f_{*}$ can be easily inferred as:

(10) $$f_{*}\;|\;x,y,x_{*}\sim N(\bar{f_{*}},\mathrm{cov}(f_{*})),$$

(11) $$\bar{f_{*}}=m+EA^{-1}(y-m),$$

(12) $$\mathrm{cov}(f_{*})=B-EA^{-1}E^{T}.$$

Accordingly, GPR can be applied for regression and prediction. Moreover, compared with single point prediction, GPR can realize interval estimation with the set CP.

In detail, the GPR prediction output includes the mean and variance of a normal distribution. So, the related confidence interval (CI) at a certain CP is $\left[\bar{f}(x_{*})-\beta \times \sqrt{\mathrm{cov}(f_{*})},\bar{f}(x_{*})+\beta \times \sqrt{\mathrm{cov}(f_{*})}\right]$, which reflects the mean range of a testing target, while PI is the interval given the noise interference—namely, $PI_{f_{*}}=[\bar{f}(x_{*})-\beta \times \sqrt{\mathrm{cov}(f_{*})+\delta_{n}},\bar{f}(x_{*})+\beta \times \sqrt{\mathrm{cov}(f_{*})+\delta_{n}}]$.

### 2.3. Prediction Interval Estimation Based on Relevance Vector Machine

Similar to the GPR model, RVM is also proposed on the basis of Bayesian framework [21], and it has the same function form as SVM, described as Equation (13):

(13) $$y(x,\omega )=\sum_{i=1}^{N}\omega_{i}K(x,x_{i})+\omega_{0},$$

where $K(x,x_{i})$ is the kernel function, $\omega_{i}$ represents the weight of the model, and $x_{i}$ is the *i*th training input with the dimension *d*. *N* is the size of training data, and ***x*** is the testing input.

Given the additive noise, the model of Equation (13) is changed to Equation (14):

(14) t=y(x,ω)+ε,

where ε is supposed to be an independent normal distribution as $\varepsilon \sim N(0,\sigma_{}^{2})$.

Based on Bayesian inference, $p(t\;|\;x)=N(t\;|\;y(x),\sigma^{2})$, the likelihood of the data set is

(15) $$p(t\;|\;\omega ,\sigma^{2})={\left(2\pi \sigma^{2}\right)}^{-N/2}\exp\{-{\left\|t-\Phi \omega \right\|}^{2}/(2\sigma^{2})\},$$

where $t={\left(t_{1},\cdot \cdot \cdot ,t_{N}\right)}^{T}$, $\omega ={\left(\omega_{0},\cdot \cdot \cdot ,\omega_{N}\right)}^{T}$, and Φ is a kernel function matrix with size N×(N+1). That is, $\Phi ={\left[\phi (x_{1}),\phi (x_{2})\cdot \cdot \cdot \phi (x_{N})\right]}^{T}$, $\phi (x_{i})=\left[1,K(x_{i},x_{1}),\cdot \cdot \cdot ,K(x_{i},x_{N})\right]$.

However, performing maximum-likelihood estimation on ω may cause the serious problem of over-fitting. So, in order to constrain these weights, Tipping defines a zero-mean Gaussian prior distribution over ω:

(16) $$p(\omega \;|\;\alpha )=\prod_{0}^{N}N(\omega_{i}\;|\;0,\alpha_{i}^{-1})=\prod_{0}^{N}\frac{\alpha_{i}}{\sqrt{2\pi }}\exp(\frac{\omega_{i}^{2}\alpha_{i}}{2}),$$

where α is the hyper-parameter vector, $\alpha =\{\alpha_{0},\alpha_{1},\cdot \cdot \cdot ,\alpha_{N}\}$. Obviously, there is a consistent one-to-one match between each weight and each hyper-parameter. Especially, the hyper-parameter value controls the influence of the prior distribution on the weights, which is also the main reason to guarantee the sparsity of the model.

To complete the specification of this hierarchical prior, we must define hyperpriors over α, as well as the noise variance $\sigma^{2}$ [21]. These quantities are examples of scale parameters, and suitable priors thereover are Gamma distributions [40]. Therefore, the posterior distributions of α and $\sigma^{2}$ are supposed to be Gamma distribution:

(17) $$\begin{array}{l} p(\alpha )=\prod_{i=0}^{N}\mathrm{Gamma}(\alpha_{i}\;|\;a,b)\\ p(\sigma^{2})=\prod_{i=0}^{N}\mathrm{Gamma}(\beta \;|\;c,d) \end{array},$$

where $\mathrm{Gamma}(\alpha_{i}\;|\;a,b)=\Gamma {\left(\alpha \right)}^{-1}b^{a}\alpha^{a-1}e^{-b\alpha }$, and $a=b=c=d=10^{-4}$.

Then, the likelihood of target output as Equation (15) can be achieved by integrating the marginal likelihood of parameters:

(18) $$p(t\;|\;\alpha ,\sigma^{2})=\int p(t\;|\;\omega ,\sigma^{2})\cdot p(\omega \;|\;\alpha )d\omega .$$

Therefore, the likelihood distribution of hyper-parameters is obtained as Equation (19):

(19) $$\begin{array}{l} p(t\;|\;\alpha ,\sigma^{2})=N(0,C)\\ =(2\pi {)}^{-N/2}{\left|\sigma^{2}I+\Phi A^{-1}\Phi^{T}\right|}^{-1/2}\exp\left\{-\frac{1}{2}t^{T}{\left(\sigma^{2}I+\Phi A^{-1}\Phi^{T}\right)}^{-1}t\right\} \end{array},$$

where $A=diag(\alpha_{0},\alpha_{1},\cdot \cdot \cdot ,\alpha_{N})$. The hyper-parameters α and $\sigma^{2}$ are estimated by iteration, which is not described in this section. Please refer to [21] to find the detailed computing process.

Suppose the new testing point is $x_{*}$, and the corresponding target is $t_{*}$. Therefore, $p(t_{*}\;|\;t)\sim N(\mu_{*},\sigma_{*}^{2})$, and the mean $\mu_{*}$ and variance $\sigma_{*}^{2}$ are given:

(20) $$\mu_{*}=\mu^{T}\phi (x_{*}),$$

(21) $$\sigma_{*}^{2}=\sigma_{MP}^{2}+\phi {\left(x_{*}\right)}^{T}\sum \phi (x_{*}),$$

where $\mu_{*}$ represents the predictive mean of $t_{*}$ and $\sigma_{*}^{2}$ indicates the predictive variance which is the combination of two variance components. In detail, $\sigma_{MP}^{2}$ is the estimated noise variance, and $\phi {\left(x_{*}\right)}^{T}\sum \phi (x_{*})$ reflects the uncertainty of weights estimation. Finally, the PI of RVM can be constructed as $\left[\mu_{*}-\beta \times \sqrt{\sigma_{*}^{2}},\mu_{*}+\beta \times \sqrt{\sigma_{*}^{2}}\right]$.

## 3. Sensing Data Anomaly Detection Based on Predicted Model with the Optimal PI

### 3.1. Analysis of PI Performance for Anomaly Detection with Different CPs

**Figure 3:**
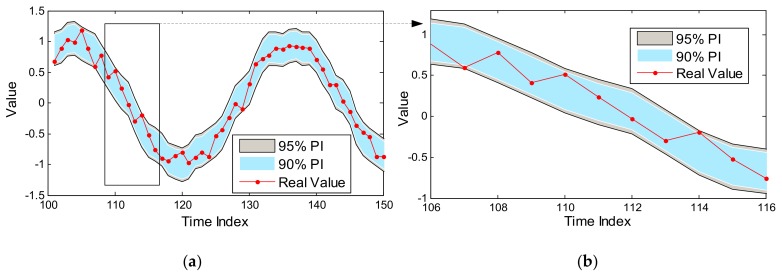
One prediction example for sine function with noise based on GPR model. (**a**) The whole prediction result; (**b**) The enlarged curve at the index from 106 to 116.

Based on the above description in Section 2, it is evident that the key step for prediction-based anomaly detection is constructing the PI, and it is sensitive to the parameters of the prediction model as well as the set CP. It must be noted that the model parameters are optimized by Bayesian framework, while the CP is set default by priori knowledge (e.g., 90% or 95%). Evidently, a higher CP has a wider PI, which will cover more training samples; on the contrary, a lower CP corresponds to a narrower PI, which may contain fewer available samples. Therefore, setting a higher CP will face the challenge of higher missing rates; otherwise, more false alarms may be produced. One GPR prediction example for sine signal with noise is shown in Figure 3.

As shown in Figure 3, PIs with two common CPs have different performances to cover the available data: 90% PI is narrower than 95% PI, which can detect more future anomalous samples. Nevertheless, 95% PI covers all samples as shown in the enlarged figure, which will cause less false alarms. It is difficult to judge whose performance is better than the other, but there is no doubt that the set CP is particularly important for constructing an effective PI. Therefore, CP should be optimized to balance the relationship between missing rate and false alarms with the available training data. In reality, anomalous samples are less or are obtained expensively, so the traditional indicator of the ROC curve which describes the relationship between sensitivity and (1-specificity) cannot be applied in this case. Thus, this work focuses on estimating the performance of PI with the available normal data and optimizing its performance to obtain an optimal CP.

### 3.2. Improved Anomaly Detection Framework with Optimal PI

**Figure 4:**
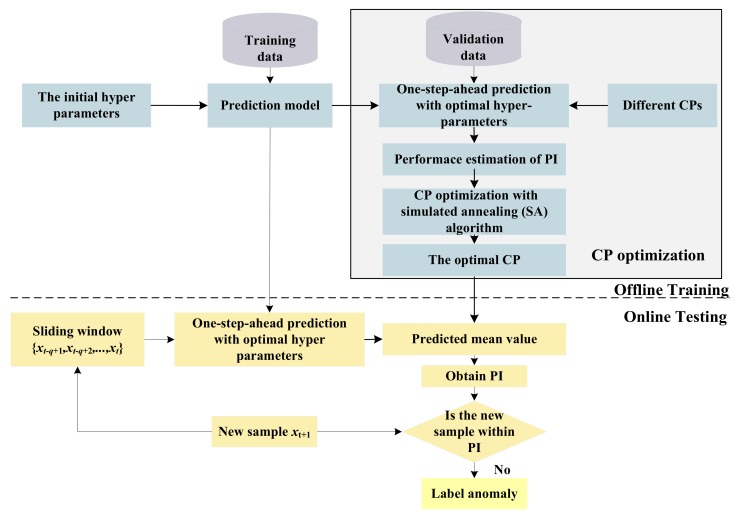
Anomaly detection framework with optimal PI.

As shown in Figure 1, the PI is computed with the set CP. Combined with the analysis of Section 3.1, the performance of anomaly detection is generally influenced by CP. Therefore, PI performance with different CPs should be assessed in the training step; especially, some optimization algorithms can be applied to determine the optimal CP. Then, this will be taken as the input parameter of the testing phase. Anomaly detection with optimal PI is realized by the framework shown in Figure 4.

As shown in Figure 4, the framework is divided into two parts: offline training and online testing.

1. Offline training

Offline training consists of two sections: hyper-parameters optimization and CP optimization.

The hyper-parameters of one prediction model are optimized according to the model requirement. In detail, GPR trains its hyper-parameters by conjugate gradient method. In addition, RVM uses expectation maximization (EM) to optimize its model parameters. These contents were introduced in Section 2.2 and Section 2.3.

CP optimization is the main focus and contribution of our work. Here the validation data set is used to determine the optimal PI. By reviewing the existing PI metrics (especially given the excellent ability of the ROC curve to estimate the performance of classification methods), this paper designs a graphic indicator (i.e., ROC-PI) to depict the trade-off between the PI width and PI coverage probability. Furthermore, the Youden index is modified to assess the detection performance with different CPs. In addition, SA is applied to optimize the modified Youden index.

Based on these two-level optimizations, the prediction model with optimal hyper-parameters and CP is realized, and will be taken as the input of online testing.

2. Online testing

At the online testing stage, a sliding window is constructed by autocorrelation analysis, and the new samples are gradually added into the sliding window. The predicted mean value and variance for a new sample are obtained by a one-step-ahead prediction model. Then, the PI is constructed effectively to label the new sample with the optimal CP. By conducting these steps repeatedly, online testing is realized continuously.

It can be summarized from Figure 4 that the CP is optimized based on historical data. Moreover, only normal samples are used to obtain the optimal CP. In other words, our proposed framework is semi-supervised, such that failure patterns are not required in the training phase. Even when some new failure patterns appear, our proposed method is also effective. This is much more meaningful for industrial applications, especially in aerospace where a large amount of normal data can be collected and the monitored data changes very slowly. In this situation, the hyper-parameters and CP optimized offline in our method has strong applicability. When the normal pattern of monitored data has strong time variability, the hyper-parameters and CP optimization are required to be updated incrementally, which is not the focus of this work.

In the following subsections, the CP optimization is described in detail, including the analysis of some PI performance indexes, the design of ROC-PI, and its optimization.

### 3.3. Performace Estimation Indexes of PI

There are currently limited indicators which have been developed to quantitatively evaluate the performance of PI [40,41]. Suppose that y is the testing target series, $y=\{y_{1},y_{2},y_{3},\ldots ,y_{n}\}$, and *n* is the testing size. For the *i*th testing input, the PI of $y_{i}$ is $\left[L_{i},U_{i}\right]$, where $L_{i}$ and $U_{i}$ are the lower and upper bounds of PI, respectively. Some related indicators are described as follows.

1. The PI coverage probability (PICP)

PICP—also called PI confidence level—is the probability that the testing targets lie within the PI provided by one prediction model [42]. PICP is derived by Equation (22):

(22) $$PICP=\frac{1}{n}\sum_{i=1}^{n}c_{i},$$

where $c_{i}$ has only two values (i.e., 0 and 1). If $y_{i}$ is within the PI of $\left[L_{i},U_{i}\right]$, $c_{i}=1$; otherwise, $c_{i}=0$. Normally, a higher PICP has a lower false rate. Ideally, PICP should be very close to 1.

2. PI normalized average width (PINAW)

PINAW, also called normalized mean prediction interval width (NMPIW), measures the wide degree of PI defined by Equation (23):

(23) $$PINAW=\frac{\sum_{i=1}^{n}\left(U_{i}-L_{i}\right)}{nr},$$

where $r=y_{\max}-y_{\min}$. PINAW is the mean of PI widths normalized by the range of testing targets. For anomaly detection, a PI which is too wide is meaningless for detecting anomalies. Another similar parameter—PI normalized root-mean-square width (PINRW) [43]—has also been designed for performance estimation, and is not described in detail in this work.

Based on the definitions of PICP and PINAM, it can be easily found that PICP and PINAW are two competing indicators, and the increase of PICP will widen the PINAW. Similarly, a wider PINAW has a better PICP value. Especially for the problem of anomaly detection, 1 - PICP is the false rate, whose best value is 0. Meanwhile, PINAW influences the detecting performance of PI. The smaller it is, the better the detecting ability it can reach. Therefore, a smaller PINAW and a larger PICP are desirable to construct PIs [42]. So, the coverage-width-based criterion (CWC) [41] is proposed to balance the relationship between PICP and PINAW, and is defined by Equation (24):

(24) $$CWC=\frac{PINAW}{\sigma (PICP,\eta ,\mu )},$$

where *σ*(·) is the sigmoidal function:

(25) $$\sigma (PICP,\eta ,\mu )=\frac{1}{1+e^{-\eta (PICP-CP)}},$$

where CP is the prior coverage probability set by users. Theoretically, PICP is unlimitedly close to or larger than CP. η is the controlling parameter which penalizes the PICP smaller than CP.

For prediction-based anomaly detection, the mean and variance of a new sample are derived by a trained prediction model. Thus, PINAW only relates to the changeable CP. Namely, we can measure the performance of PI by CP and PICP. Although CWC can balance the relationship between the width of PI and PICP, it is not effective for anomaly detection. For example, the optional range of CP changes from 90% to 100%. Normally, 90% PI has a better CWC, because 90% CP has a smaller PINAW. At the same time, the PICP corresponding to 90% CP is generally larger than 90% (the increasing speed of PICP is usually reduced with the increasing CP). In this case, the CWC is invalid to determine the optimal CP of anomaly detection. Therefore, the definition of the ROC curve is applied as the basis of this work.

### 3.4. Receiver Operating Characteristic (ROC) Curve of Prediction Interval 

**Figure 5:**
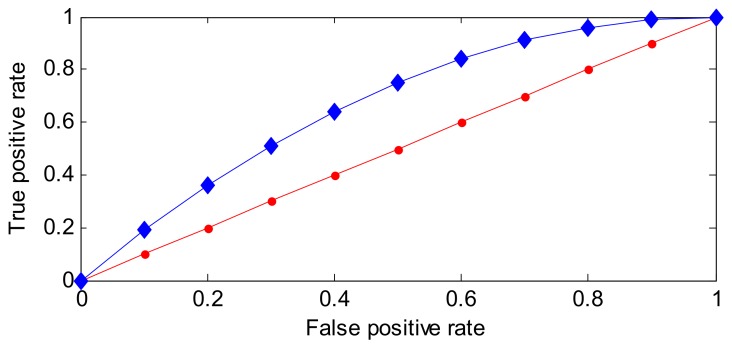
One example of a receiver operating characteristic (ROC) curve.

A ROC [33,44] curve is a plot that depicts the trade-off between the sensitivity and (1-specificity) across a series of cut-off points. One example of a ROC curve is shown in Figure 5.

As shown in Figure 5, the properties of a ROC curve can be concluded as follows.

1. The horizontal axis reflects the false positive rate (FPR), which indicates the positive samples labeled negative. FPR ranges from 0 to 1. Ideally, FPR equals 0.
2. The vertical axis is the true positive rate (TPR), which also ranges from 0 to 1. Ideally, TPR equals 1.
3. With the ROC curve, two or more classification methods can be visually compared in one figure.
4. Ideally, TPR = 1, FPR = 0, and the more ROC curves closer to the (0, 1) point, the better the performance is.

Actually, there are not enough anomalous samples in the training step, so the ROC curve cannot be applied directly. Therefore, in this paper, a new indicator (i.e., ROC-PI) is designed on the basis of the ROC curve, where the original vertical axis of sensitivity is tuned to PICP (which indicates the detection rate for the testing samples) and the horizontal axis is changed to these set CPs (which represents the performance of PINAW).

**Figure 6:**
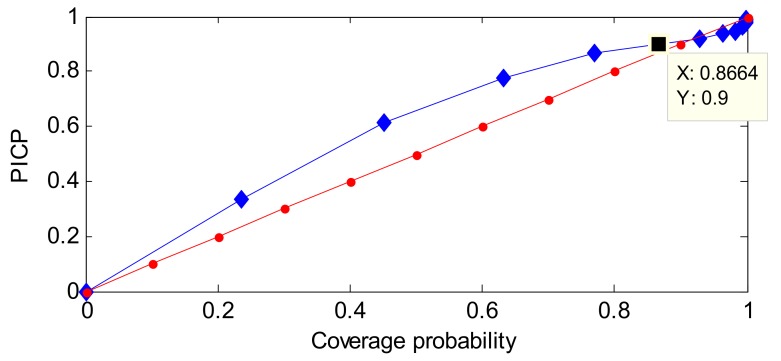
One example of receiver operating characteristic of PI (ROC-PI) based on the relevance vector machine (RVM) method. PICP: PI coverage probability.

One example of a ROC-PI curve based on an RVM model is given in Figure 6.

As shown in Figure 6, the properties of ROC-PI are listed as follows.

1. PICP is the rate that normal data are labeled normal, and a larger PICP has a better performance. CP means the set priori coverage probability of the normal distribution. If the prediction model can describe the distribution of each new sample well, PICP should be greater than or equal to CP—ideally, the point close to (1, 1) has a better performance.
2. In general, at the initial stage of CP growth, more data will be covered. So, PICP is larger than CP. However, at the late stage of CP growth, there are fewer points beyond the corresponding PI, the growth rate of CP will be faster than PICP’s. Therefore, the point where PICP equals CP has a better performance within the effective range of CP.
3. The ROC-PI can also be applied to estimate the performance of different models than the ROC curve. In addition, the area under the ROC-PI curve has a similar meaning to area under the curve (AUC).The diagonal line describes that the PICP values equals CP values. In general, the ROC-PI is above the diagonal line.

The Youden index is defined as the difference between TPR and FPR, and has been applied to select the optimal point in the ROC curve [45]. Based on the above analysis of ROC and ROC-PI curves, a similar performance can be concluded. So, the Youden index may be modified to adapt to this work. 

On the basis of the Youden index definition, the difference between PICP and CP can also be used as a performance estimator. It is worth noting that CP is the set coverage probability of PI, and PICP reflects the posterior coverage probability of PI. Ideally, PICP should be very close to or greater than CP. As shown in Figure 6, with increasing CP, the difference between CP and PICP becomes larger. In other words, most of the available samples are gradually covered by the constructed PI. Conversely, the difference becomes smaller at the late stage of CP growth, and even becomes negative. The reason is that the PICP increase will cause a significant increase of PI. Accordingly, the optimal CP has the minimum absolute value of the difference between PICP and CP. Namely, the evaluation function of PI performance is the modified Youden Index defined by Equation (26):

(26) Y=|PICP−CP|.

### 3.5. Optimize the Coverage Probability of PI

For the probability prediction models (e.g., GPR and RVM), the prediction output is the series of confidence values ordered from small to large, and the PI with confidence value α is defined as:

(27) $$PI_{\alpha }=\gamma^{1-\alpha /2}-\gamma^{\alpha /2},$$

where γ is the quantile value corresponding to its superscript.

Then, the normal range for a new sample can be described by $PI_{\alpha }$. For normal distributions, CP = 1−α. As PI is an estimate of an interval within which one or more future observations will fall given what has already been observed, $PI_{\alpha }$ indicates that a new observation will fall into the PI with the probability of 1−α. For example, α = 5%, $PI_{0.05}$ means that a new observation may fall into the PI with a probability of 95%. Obviously, with decreasing α, the PI will become wider, and the probability of the PI covering a new observation will become larger. 

In this work, ROC-PI is proposed to describe the performance of PI under different CPs. Moreover, the Youden index is modified to optimize the CP of PI. Generally, we can examine the ROC-PI curve to select the optimal CP. However, it needs to compute several PICP values under a series of CPs. Actually, a small CP cannot obtain a good PICP with the assumption that the prediction model can describe the distribution of new samples well. Furthermore, given that the modified Youden index is not an analytic formula, it cannot be optimized by gradient descent method. Therefore, the CP optimization is realized by SA optimization technique in this work, which has been utilized to solve this type of optimization problem [46]. The SA algorithm randomly explores the neighborhood of the current solution, seeking a better solution which escapes from local minima with the probability of accepting a new solution that influences the cost function. Additionally, the probability is controlled by a parameter called the cooling temperature.

**Figure 7:**
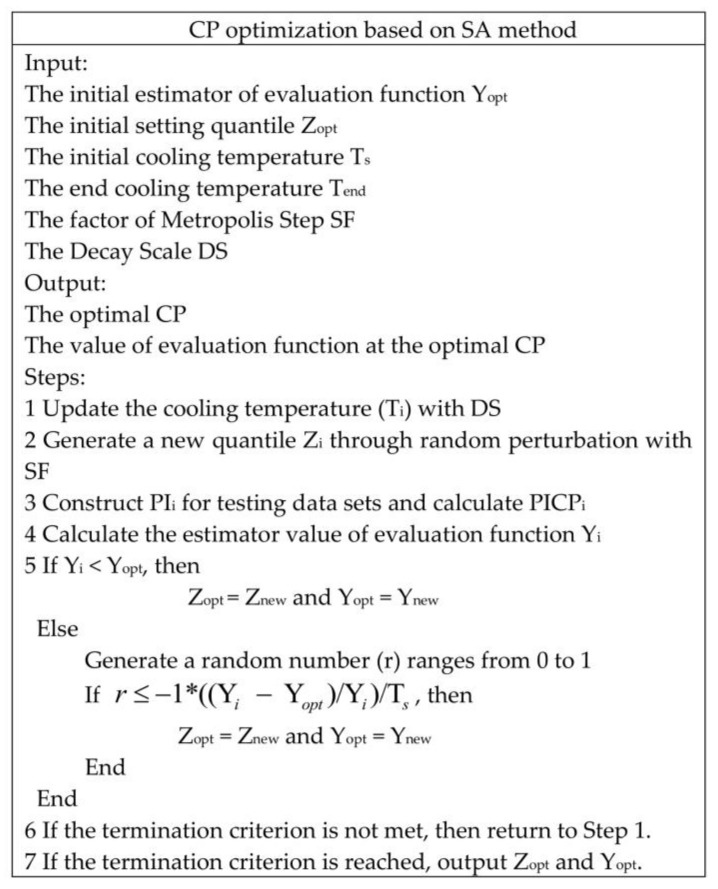
CP optimization based on simulated annealing (SA).

The training data set is divided into two sets: the training set and the validation set. They are applied to training the prediction model and optimizing the CP respectively. The detailed training procedures of GPR and RVM models are given in Section 2.2 and Section 2.3. So, this section only gives the pseudocode of CP optimization with the SA algorithm, as shown in Figure 7.

In Figure 7, the evaluation function is the modified Youden index. Since the PI is described by a normal distribution, one quantile corresponds to a specific CP; e.g., 1.96 is the quantile corresponding to a CP of 95%, and the corresponding CP for 1.65 is 90%. Thus, we can search the quantile that minimizes the evaluation function. Then, the CP related to this quantile is optimal for our task. The cooling temperature is set to allow uphill movement in the early iterations of the optimization algorithm which ranges from T_s_ and T_end_, and the decay scale (DS) controls the cooling speed. In addition, the step factor of Metropolis (SF) is applied to generate a new quantile through random perturbation. At each iteration, a new quantile is generated within the setting range. PIs are constructed for each new quantile, and the optimal CP—together with the minimum value of the modified Youden index—will be the output of this optimization algorithm.

## 4. Experimental Results and Analysis

In this paper, the experimental validation is performed in two aspects. Firstly, two simulated data sets with injected anomalous samples are applied to measure the anomaly detection performance of this proposed method. Then, some typical telemetry series are applied to verify the practicality and effectiveness of our method in real applications.

The metrics are false positive rate (*FPR*), false negative ratio (*FNR*), and accuracy (*ACC*).

1. *FPR*

*FPR* is the ratio that the normal data is falsely detected and rejected.

(28) $$FPR=\frac{FP}{FP+TN}\times 100\%,$$

where FP (false positive) represents the amount of normal data samples regarded as anomalies, and *FP* + *TN* (true negative) is the sum of the normal data samples.

2. *FNR*

*FNR* is the ratio that the abnormal data is detected in error and accepted.

(29) $$FNR=\frac{FN}{TP+FN}\times 100\%$$

where *FN* indicates the number of abnormal data points detected as normal points, and *TP* + *FN* refers to the number of the anomalous data points. Normally, smaller *FNR* and *FPR* implies better performance of anomaly detection.

Generally, the classification of normal and anomalous are unbalanced. Moreover, *FNR* and *FPR* are contradictory. In order to estimate the performance by one indicator effectively, *ACC* is utilized and is defined by Equation (30):

(30) $$ACC=\frac{TP+TN}{FP+FN+TN+TP}\times 100\%,$$

where *FP* + *FN* + *TN* + *TP* is the amount of all data detected, and *TP* + *TN* is the amount detected correctly. Namely, accuracy (*ACC*) is the ratio of the correctly detected normal data and anomalous data in the total detected data.

### 4.1. Experiments on Simulated Data Sets

In order to evaluate the anomaly detection performance, two typical series of Keogh_data and Ma_data are applied in this subsection. With the certain amount and location of the injected anomalies, the quantitative evaluation results can be given.

Keogh_Data is a simulated data set which has been utilized to test three anomaly detection algorithms referred to as IMM, TSA-Tree, and Tarzan in [47]. Moreover, many studies have introduced this data set to verify the algorithm performance [48,49]. Therefore, two types of abnormal series injected into Keogh_Data are applied to estimate the performance of our proposed method, and they are named Keogh_Data 1 and Keogh_Data 2, respectively.

Keogh_Data 1 is generated by Equation (31):

(31) $$\begin{array}{l} Y_{1}=\sin(\frac{50\pi }{N}t)+n(t)\\ Y_{2}=\sin(\frac{50\pi }{N}t)+n(t)+e_{1}(t) \end{array},$$

where t=1,2,3…..N, N=800, and n(t) is the white Gaussian noise with zero mean and standard variance 0.1. In addition, $e_{1}(t)$ reflects the customized abnormal mode, which is defined as Equation (32):

(32) $$e_{1}(t)=\{\begin{array}{c} \sin(\frac{25\pi }{N}t),\;t\in [400,432]\\ \;0,\;otherwise \end{array}.$$

Keogh_Data 2 is defined by Equation (33):

(33) $$\begin{array}{l} Y_{1}=\sin(\frac{50\pi }{N}t)+n(t)\\ Y_{2}=\sin(\frac{50\pi }{N}t)+n(t)+e_{2}(t) \end{array},$$

where $e_{2}(t)$ is also the injected abnormal mode which is defined by Equation (34):

(34) $$e_{2}(t)=\{\begin{array}{c} \sin(\frac{75\pi }{N}t)-\sin(\frac{50\pi }{N}t),\;t\in [400,432]\\ \;0,\;otherwise \end{array}.$$

Additionally, Ma_Data is generated from a stochastic process which was used to test SVR algorithm [50].

(35) $$\begin{array}{l} Y_{1}=\sin(\frac{40\pi }{N}t)+n(t)\\ Y_{2}=\sin(\frac{40\pi }{N}t)+n(t)+e_{3}(t) \end{array},$$

where n(t) is also the white Gaussian noise with zero mean and standard variance 0.1 and $e_{3}(t)$ is the simulated white Gaussian noise with zero mean and variance 0.5.

**Figure 8:**
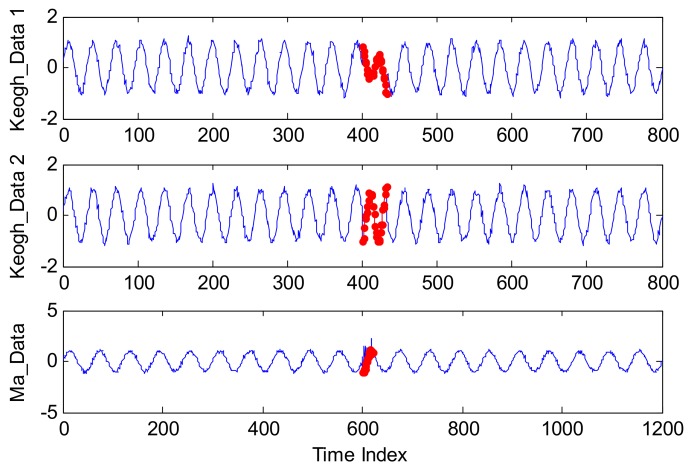
Time series of Keogh_Data 1, Keogh_Data 2, and Ma_Data.

Some examples of Keogh_Data 1, Keogh_Data 2, and Ma_Data are shown in Figure 8.

In Figure 8, for each simulated series, the blue line represents the normal points generated by the equation of Y1, while the points labeled by a red star are anomalous as defined by the equation of Y2.

The quantitative results based on the optimal CP as well as the default CP (i.e., 90% and 95%) are shown in Table 1 and Table 2, where the metrics are the mean value of ten random experiments.

As shown in Table 1 and Table 2, the PIs with these optimal CPs have a better performance for detecting anomalies. For example, for Keogh_Data1, the optimal CP for RVM model is 97.16%, the ACC of which is 94.8%. Meanwhile, the PIs with the default values of 90.00% and 95.00% are 87.20% and 91.40%, respectively (in order to ensure consistency in the number of significant digits, we have added several invalid zero at the end of the related numbers), and the improvements are 8.72% and 3.72%, respectively. Correspondingly, for Keogh_Data2, the optimal CP is 99.93% for the GPR model, the ACC of which is 96.60%. Correspondingly, the PI with the default value of 90.00% and 95.00% are 94.20% and 95.80%, respectively. It is noted that for Ma_Data, the optimal CPs are both 95.00% for GPR and RVM models. In other words, for different series, in order to obtain the better detection performance, the CP should be optimized rather than setting a default value.

### 4.2. Experiments on Normal Telemetry Series

When a spacecraft works on orbit, some sensor-based monitoring information will be encoded and transmitted into ground center. This is the only basis for the ground monitoring personnel to judge the working performance of on-orbit spacecraft. Therefore, anomaly detection of these series is very meaningful for enhancing the reliability and safety of the spacecraft systems. Given that the orbit of spacecraft is generally regular, together with the regular change of system working mode, some telemetry series show a pseudo-periodic property. So, in this subsection, some typical satellite telemetry series from power subsystems are applied to verify the validity of our work. As we made an analysis of the ROC-PI curve in Section 3.4, generally, PICP increases sharply at the beginning of CP while the rate of increase becomes slower at larger CP. This analysis allows us to determine the optimal CP with the smallest difference of PICP and CP. Therefore, some normal satellite series are first applied to test the effectiveness of our analysis on the ROC-PI curves. We also use the ROC-PI curves of normal telemetry series to validate the effectiveness of realizing the CP optimization with SA method.

**Figure 9:**
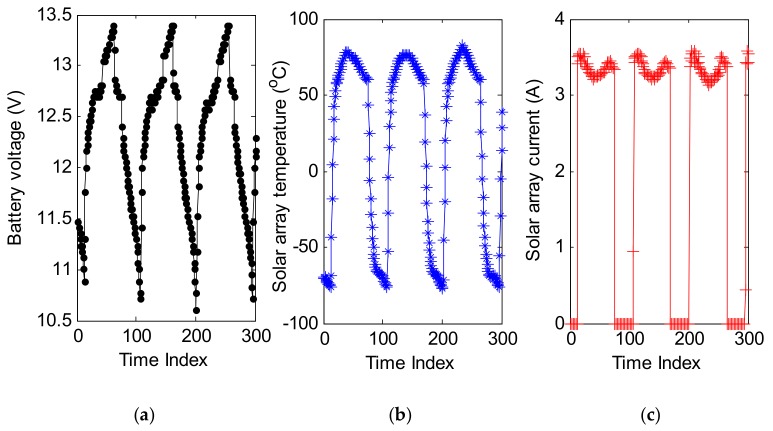
Three types of telemetry series. (**a**) Battery voltage; (**b**) Solar array temperature; (**c**) Solar array current.

The power subsystem of a satellite is mainly composed of a solar array-battery system, a charge regulating circuit, a discharge regulating circuit, and a shunt regulation circuit. The typical monitoring types are generally current, voltage, and temperature. So, three types of satellite telemetry series (i.e., solar array current, battery voltage, and solar array temperature from power subsystem) were selected as the test sequences, and are shown in Figure 9. These series were resampled by one minute.

The training data size for the three series was 1000, and the embedded dimension was determined by autocorrelation analysis to construct the input matrix. Due to the periodic property of these series, the validation data set was less likely to be generated by resampling method. Thus, we merely selected the last 500 samples as the validation set. The size of testing data was set to 2000.

The covariance function of GPR is a square exponential function, which is very common for performing prediction as defined by Equation (3). The hyper-parameters of the mean function were set to zero. In addition, the initial hyper-parameters in the covariance function were set to random values from 0 to 1. For the RVM model, the kernel function is a Gaussian kernel function, and the width of the Gaussian function was 8. Moreover, the estimated hyper-parameters were (1/*N*)^2^, where *N* is the training size.

**Figure 10:**
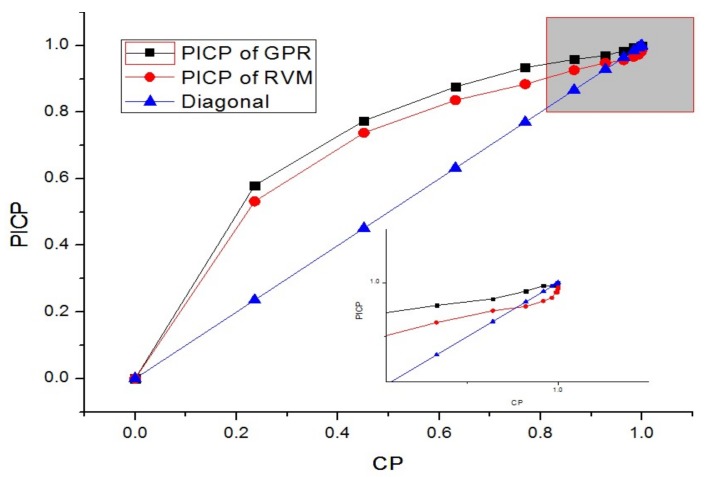
ROC-PI curves of RVM and GPR for the solar array current series.

**Figure 11:**
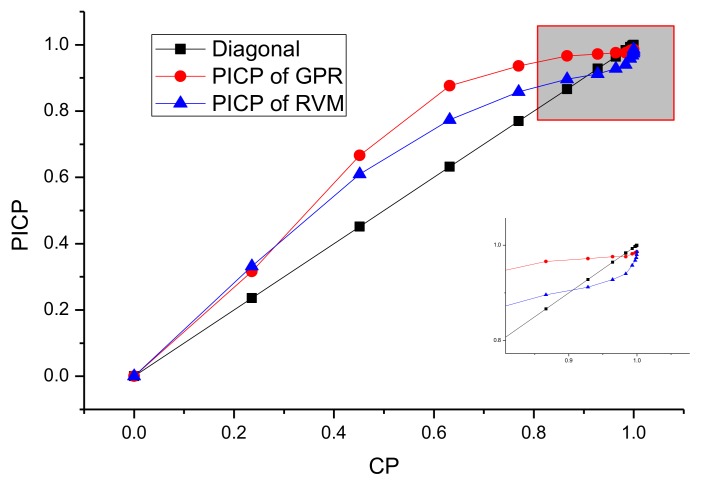
ROC-PI curves of RVM and GPR for battery voltage series.

**Figure 12:**
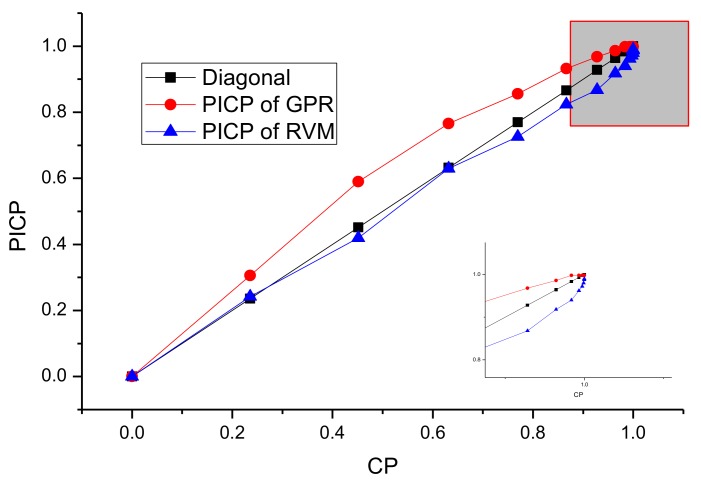
ROC-PI curves of RVM and GPR for solar array temperature series.

For the SA algorithm, the quantile of prediction ranges from 1.4 to 4, which corresponds to the CP from 90 to 99%. The initial quantile (Z_opt_) is 1.96, whose corresponding CP is 95%. The step factor of Metropolis (SF) is 0.25, the decay factor (DS) is 0.85. The initial cooling temperature (T_s_) is 15, and the end cooling temperature (T_end_) is set to 1. For RVM and GPR models, the ROC-PI curves with different CPs for these three-telemetry series are shown in Figure 10, Figure 11 and Figure 12.

As shown in Figure 10, Figure 11 and Figure 12, the optimal PI are different for various data sets. Moreover, compared with GPR, the PI of RVM is relatively narrow. The PICP generally increases more quickly at the start of CP, while it has a slower pace at the end of CP (as shown in Figure 10 and Figure 11). However, for Figure 12, the PICP of RVM is smaller than CP at the CP ranging from 90% to 99%. This indicates that the performance of PI constructed by RVM is relatively poor for solar array temperature. Under the circumstances, the CP optimization by SA will select a better CP to improve the PI performance of the RVM model. The optimal CPs for different series based on SA are given in Table 3.

Compared with the enlarged figures from Figure 10, Figure 11 and Figure 12, the CP optimized by SA keeps high consistency with the optimal values intuitively shown in ROC-PI curves. The CP optimization is to keep a lower false rate with a higher detection performance given what has already been observed. Therefore, it can provide an effective PI for the following anomaly detection.

### 4.3. Experiments on Telemetry Series with Anomalies

**Figure 13:**
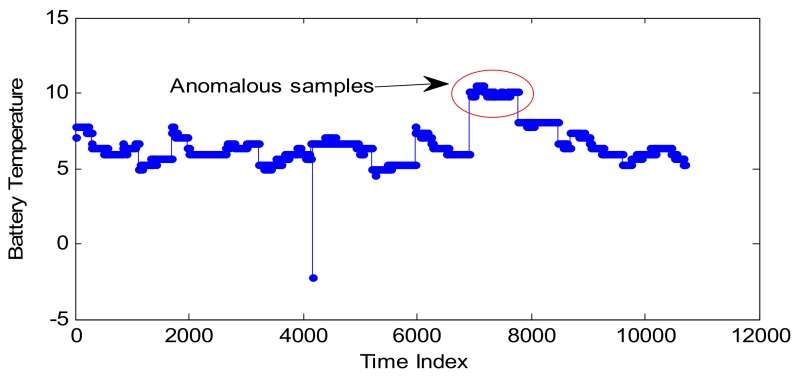
Battery temperature series with anomalous samples.

In Section 4.2, the performance of CP optimization based on SA is verified by three normal series. However, there are no anomalous samples within these three series. In order to verify the anomaly detection performance, one telemetry series—collected by a temperature sensor from another satellite—is introduced as the test series, which is shown in Figure 13. Some abnormal samples appear on April 11th. It is noted that these anomalous samples are not much larger, so they cannot be effectively detected by fixed threshold, which are usually set much bigger than normal samples.

**Figure 14:**
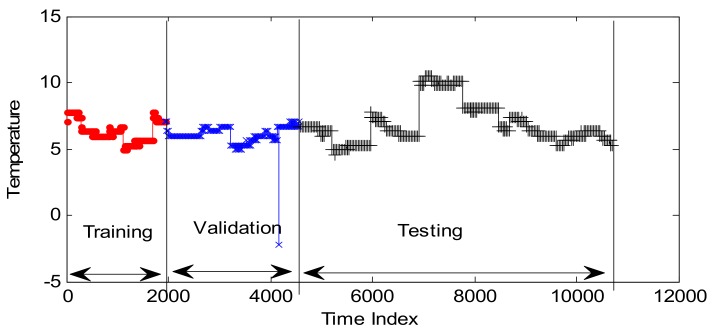
The training set, validation set, and testing set.

GPR and RVM models are also applied to detect the anomalies with the optimal CPs. Here the embedded dimension is 37, determined by autocorrelation analysis method. Other parameters are consistent with the parameter setting in Section 4.1. The training set contains the samples from April 8th and April 9th, and the validation set includes the points from April 10th. The real test data set is set to the samples from April 11th to 13th. The detailed design is shown in Figure 14.

**Figure 15:**
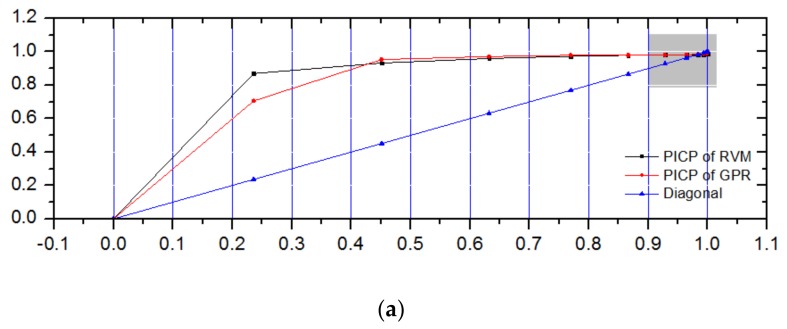

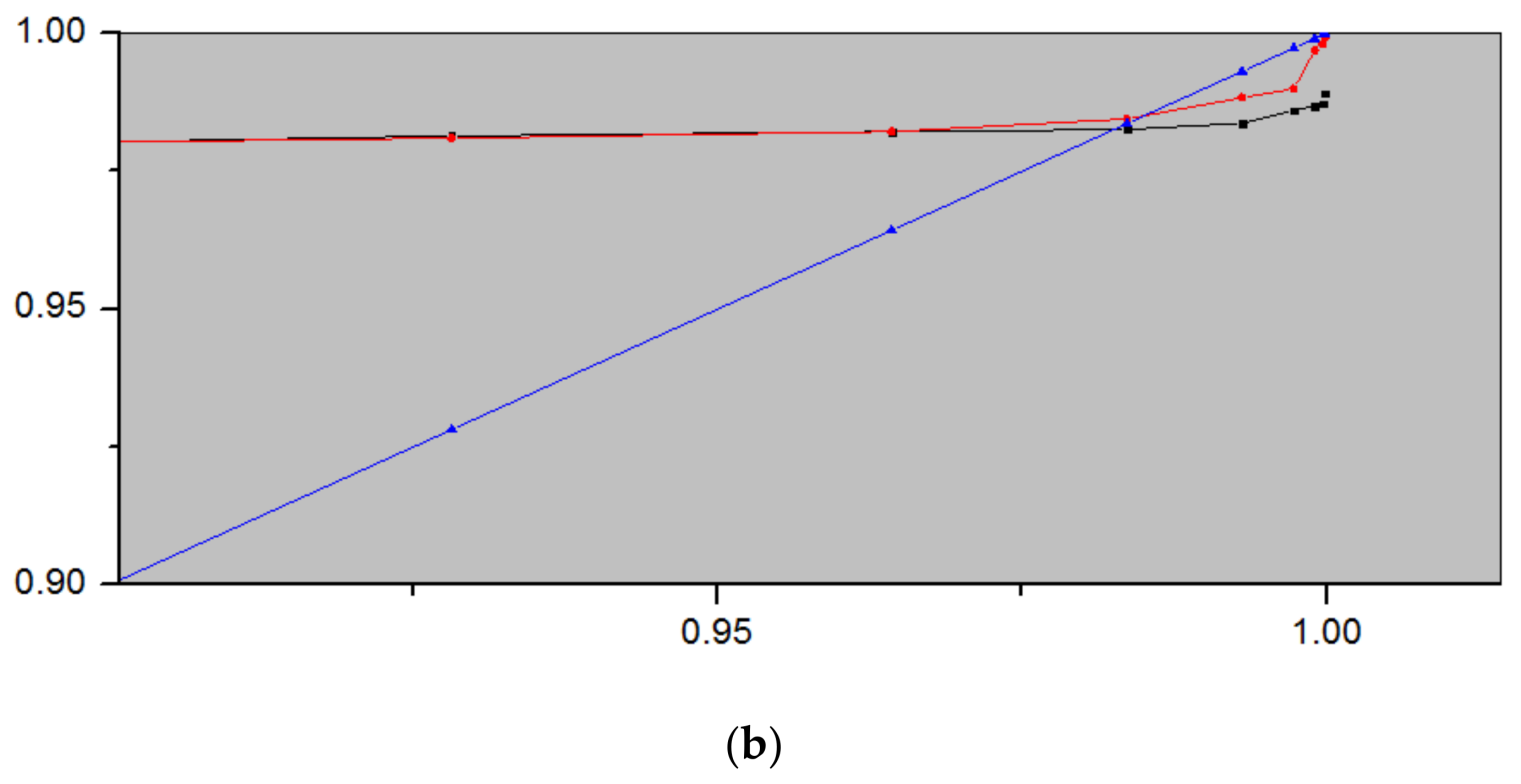
ROC-PI curves of GPR and RVM models for battery telemetry series. (**a**) ROC-PI curves of GPR and RVM models; (**b**) The enlarged figure of ROC-PI curve.

The optimal CPs of GPR and RVM based on SA are 98.41% and 97.30%, respectively. In order to make a comparison, we also depict the ROC-PI curves, which are shown in Figure 15. 

As shown in Figure 15, the PICP of RVM and GPR increase sharply at a smaller CP; for example, the PICP of RVM reaches 0.7 at a CP of 0.2. Meanwhile, as the CP increases, the PICP increases slowly. This case verifies the effectiveness of our analysis on ROC-PI. Moreover, in our proposed method, the intersection of ROC-PI and line of equality corresponds to the optimized CP, which can be applied to realize anomaly detection. For Figure 15, the intersections of ROC-PI curves for GPR and RVM models keep high insistence with the optimal CPs of 98.41% and 97.30% derived by the SA algorithm, respectively. Thus, our proposed method is effective in obtaining the optimal CP to realize anomaly detection.

**Figure 16:**
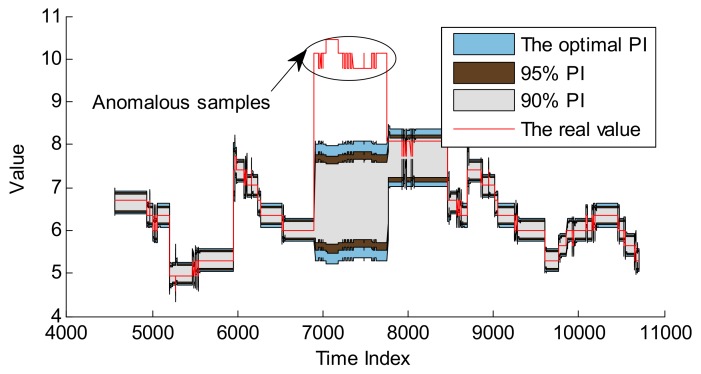
Anomaly detection for battery temperature series based on GPR model.

**Figure 17:**
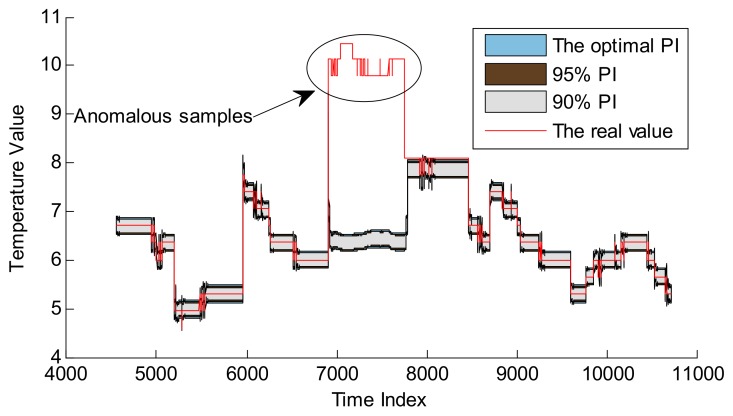
Anomaly detection for battery temperature series based on RVM model.

Based on the hyper-parameters optimization of GPR and RVM, as well as CP optimization, these two models can be applied to realize the following anomaly detection. The detection results are shown in Figure 16 and Figure 17, and the quantitative results are given in Table 4.

As shown in Figure 15, it is obvious that at the start of CP, the PICP increases quickly. However, the PICP increases at a slower step when the CP is close to 1. Especially, the optimal CP of GPR is larger than the optimal CP of RVM (as shown in Figure 15), which is consistent with the result of CP optimization by SA method. 

Compared with Figure 16 and Figure 17, GPR and RVM models can effectively detect these anomalous samples, and the PI with optimal CP is larger than that with default CPs which can be applied to realize better anomaly detection. The results of detecting anomalies in Table 4 can better describe the superiority of PI with the optimal CP. For example, the *ACC* of the GPR model with the CP of 98.41% is 98.96%, which is better than 98.33% and 98.75% for the CPs of 90% and 95%. A similar conclusion can be made for the RVM model. It is noted that the improvement is not evident, as these anomalous samples are relatively larger than normal data. Moreover, one evident difference is that the PIs of GPR at the anomalous indexes become larger than normal while the PIs of RVM are not influenced by these anomalous samples. The main reason is that the RVM model computes the new prediction by the projection of the original input into the relevance vector space. The GPR model directly computes the covariance of the testing set with the training set to make predictions. This means that for longer anomalous fragments, the RVM model is more robust than the GPR model. On the other hand, the GPR model is more effective if there are no anomalous samples. It is very meaningful that both GPR and RVM models can detect the real anomalous samples. Based on these warning alarms, the ground personnel can set telecommand to moderate the temperature to improve the reliability of the battery in case of causing fatal failure.

### 4.4. Results Analysis and Discussion

In the first experiment, it is noted that the missing rate of each method—even with the optimal CPs—is relatively high. The main reason is that we label the single point, not the whole fragment. In the second experiment, three telemetry series are applied to evaluate the performance of optimizing the CPs based on SA. The optimal CP keeps high consistency with the ROC-PI graphical indicator. Moreover, based on the ROC-PI curve, it is shown that the optimal CP is different for various series. Namely, the default CP cannot adapt to any series with which the detecting performance will be influenced without doubt.

In the third experiment, the anomaly detection for a real sensor series is realized. The appearing anomalous samples are larger than normal, so the detection rate is 100% with different CPs. However, the detection with optimal CP can obtain better performance with relatively lower FNRs. Thus, the PI with optimal CP has better extensibility for the unknown testing samples. Obviously, the improvement of *ACC* is smaller than the experiment on simulated data sets, with the main reason being that these anomalous samples are larger than normal.

Moreover, in this work, the distribution of the simulated data sets and the telemetry series are similar for the training data sets, validation sets, and testing sets, so the validation sets are not resampled by some methods (e.g., cross-validation, hold out, or bootstrapping). In other words, if the data sets are insufficient or imbalanced, some resampling methods should be applied to generate the validation set.

For the ROC-PI curve, two types of cases are not discussed in this work; namely, the PICP reaches 1 at a relatively smaller CP, and the CP reaches 1 with a smaller PICP. These two cases indicate that the PI generated by the prediction model cannot describe the distribution of the real value, and it is not consistent with our hypothesis. In one case, the smallest CP with PICP which equals 1 is the optimal CP, and in the other case, the prediction model cannot be applied for the detecting application. These phenomena may happen in the real applications, which should be processed especially.

## 5. Conclusions 

The contributions of this work can be concluded as: (1) The graphical indicator ROC-PI is first proposed to measure the model performance with different CPs, which depicts the trade-offs between the PI width and PI coverage probability across a series of cut-off points; (2) CP is optimized by the modified Youden index with SA algorithm; (3) The improved anomaly detection method based on probability prediction model is utilized to achieve abnormal detection; (4) The detecting performance of GPR and RVM is compared and analyzed; (5) Actual in-orbit satellite telemetry data are labelled effectively by GPR and RVM models. 

There is also some work which needs to be conducted in the future: (1) More prediction models can be applied to realize anomaly detection to demonstrate the universality of this method. (2) The hyper-parameters within the prediction model should be optimized with the cost function of anomaly detection. (3) Especially, anomaly detection for other types of unmanned aerial vehicle should be considered in the future. 

## Figures and Tables

**Table 1:** Anomaly detection with different CPs based on relevance vector machine (RVM) model. *ACC*: accuracy; *FNR*: false negative rate; *FPR*: false positive rate.

Data set	CP	*FNR*	*FPR*	*ACC*
Keogh_Data 1	90.00%	21.77%	12.21%	87.20%
	95.00%	27.27%	7.28%	91.40%
	97.16% (optimal)	27.27%	3.64%	94.80%
Keogh_Data 2	90.00%	6.06%	12.42%	88.00%
	95.00%	12.12%	7.28%	92.40%
	97.10% (optimal)	15.15%	3.64%	95.60%
Ma_Data	90.00%	23.81%	10.60%	89.00%
	95.00% (optimal)	33.33%	5.45%	93.71%

**Table 2:** Anomaly detection with different CPs based on Gaussian process regression (GPR) model.

Data set	CP	*FNR*	*FPR*	*ACC*
Keogh_Data 1	90.00%	33.33%	4.93%	93.20%
	95.00% (optimal)	33.33%	2.78%	95.20%
Keogh_Data 2	90.00%	12.12%	3.64%	94.20%
	95.00%	12.12%	5.35%	95.80%
	99.93% (optimal)	18.78%	2.36%	96.60%
Ma_Data	90.00%	28.57%	10.60%	88.86%
	95.00% (optimal)	33.33%	5.45%	93.29%

**Table 3:** The optimal CP for telemetry series based on SA.

Method	Solar Array Current	Battery Voltage	Solar Array Temperature
GPR	99.35%	97.66%	99.78%
RVM	95.00%	90.67%	99.17%

**Table 4:** Anomaly detection with different CPs.

Algorithm	CP	*FPR*	*FNR*	*ACC*
GPR	90.00%	1.94%	0.00%	98.33%
	95.00%	1.45%	0.00%	98.75%
	98.41% (optimal)	1.21%	0.00%	98.96%
RVM	90.00%	15.00%	0.00%	87.08%
	95.00%	14.60%	0.00%	87.42%
	97.30% (optimal)	14.04%	0.00%	87.91%
